# Morphology-Controlled γ-Alumina Adsorbents: Fluoride Removal Performance and Cyclic Regeneration Stability in Aqueous Media

**DOI:** 10.3390/molecules31152634

**Published:** 2026-07-29

**Authors:** Kexin Ge, Chunlin Zhao, Dehao Zhang, Miao Yi, Shuaiqi Chen, Boning Jiang, Xuhui Wang, Xiangyu Xu, Jiaqing Song

**Affiliations:** 1 College of Chemistry, Beijing University of Chemical Technology, Beijing 100029, China; 2024210791@mail.buct.edu.cn (K.G.); 2022430033@mail.buct.edu.cn (S.C.); 2023400285@mail.buct.edu.cn (B.J.); songjq@mail.buct.edu.cn (J.S.); 2 State Key Laboratory of Chemical Resource Engineering, Beijing University of Chemical Technology, Beijing 100029, China; 3 Beijing Tiandun New Materials Technology Co., Ltd., Beijing 100094, China; zhaochunlin202305@163.com; 4 SINOPEC (Beijing) Research Institute of Chemical Industry Co., Ltd., Beijing 100013, China; 18811553133@163.com; 5 College of Artificial Intelligence and Information Engineering, Yi Chun University, Yichun 336000, China; styimiao@163.com; 6 Sinopec Catalyst Co., Ltd., Beijing 100176, China; xuhuiwang@126.com

**Keywords:** morphology regulation, γ-alumina, fluoride removal, water remediation, adsorption, cyclic regeneration

## Abstract

Excessive fluoride in groundwater and industrial wastewater poses serious risks to human health. Although commercial γ-alumina is a low-cost adsorbent recommended for defluoridation, its limited adsorption capacity and poor regeneration stability hinder its practical application. In this work, three template-free γ-alumina materials with distinct morphologies (nanosheets, rhombic flakes, and nanofibers) were synthesized by a mild hydrothermal method and systematically compared with commercial granular γ-alumina. To isolate the effect of morphology, all four γ-alumina adsorbents were prepared with comparable BET surface areas (180–230 m^2^·g^−1^). Their structural, morphological, and surface properties were comprehensively characterized, and their fluoride adsorption performance, adsorption mechanism, and regeneration behavior were systematically evaluated. Despite their similar BET surface areas, the four samples exhibited markedly different adsorption performances. At an initial fluoride concentration of 600 mg·L^−1^, the adsorption capacity followed the order: nanosheets (87.2 mg·g^−1^) > nanofibers (49.0 mg·g^−1^) > rhombic flakes (46.1 mg·g^−1^) > commercial alumina (35.0 mg·g^−1^). The superior adsorption performance of the nanosheet morphology suggests that the accessibility and local chemical environment of surface hydroxyl species, rather than their total abundance alone, play an important role in fluoride adsorption. In contrast, nanofibrous XW-600 exhibited superior resistance to repeated acid–alkali regeneration, preserving its crystal structure and fibrous morphology while maintaining the highest residual adsorption capacity among the synthesized alumina samples after five regeneration cycles. Overall, the results suggest that alumina morphology strongly influences fluoride adsorption performance and regeneration stability. This work provides a simple, template-free strategy for preparing morphology-controlled γ-alumina and offers new insights into the relationship between alumina morphology and fluoride adsorption, providing guidance for the rational design of high-performance, regenerable alumina adsorbents.

## 1. Introduction

Fluoride is an essential trace element for human dental health at an optimal concentration of ~0.7 mg∙L^−1^. However, long-term intake of drinking water with fluoride exceeding the WHO permissible limit (1.5 mg∙L^−1^) causes irreversible dental fluorosis, skeletal fluorosis, and severe damage to endocrine organs, thyroids, and livers [1,2]. Globally, over 200 million people rely on high-fluoride groundwater as their daily drinking source, and more than 70 million individuals suffer from fluoride-induced chronic diseases [3]. To mitigate fluoride contamination in aqueous media, multiple treatment technologies have been developed, including adsorption [3,4,5,6,7,8], coagulation–sedimentation [9], electrocoagulation [10], and membrane filtration (reverse osmosis and electrodialysis) [11,12,13]. Among these approaches, activated alumina adsorption is officially recommended by the WHO and U.S. environmental authorities as a low-cost, high-efficiency defluoridation technique suitable for decentralized drinking water purification and industrial wastewater treatment [14,15,16,17,18,19,20]. Unfortunately, conventional granular alumina adsorbents exhibit unsatisfactory saturated fluoride adsorption capacities, which drastically increase material consumption and operational costs in practical wastewater treatment [15].

Previous research generally attributed alumina adsorption capacity solely to large specific surface areas, which provide abundant surface hydroxyl active sites for fluoride binding [15]. Two mainstream strategies—surface chemical modification and morphological engineering—have been adopted to boost the defluoridation performance of alumina [21,22,23,24]. A diverse library of nanostructured alumina morphologies, including nanoribbons, nanotubes, nanorods, nanofibers, and nanosheets, has been successfully synthesized in prior studies [25,26,27,28,29,30]. Distinct exposed crystal facets induced by varied microstructures endow alumina with divergent surface chemical properties; yet systematic comparative investigations correlating micromorphology with fluoride adsorption behavior remain scarce.

In our previous work, template-free monolayer boehmite with a BET surface area of 789.4 m^2^·g^−1^ was successfully synthesized, and the corresponding γ-alumina exhibited a fluoride adsorption capacity of 67.6 mg·g^−1^ [23]. Building on this foundation, the present work fabricates three boehmite precursors with nanosheet, rhombic flake, and nanofibrous architectures via a template-free mild hydrothermal method by tuning the synthesis parameters [23,29,30]. After calcination at 600 °C, four γ-alumina adsorbents, including a commercial granular alumina and three laboratory-synthesized samples with distinct morphologies, were obtained. To isolate the intrinsic effect of morphology on fluoride adsorption, the BET surface areas of the four adsorbents were intentionally controlled to be comparable within this study, thereby minimizing the influence of specific surface area. Systematic batch adsorption experiments and multi-cycle regeneration tests were subsequently performed to evaluate the effects of morphology on fluoride adsorption performance, adsorption kinetics, regeneration behavior, and structural stability under alternating acid–alkali conditions. Through this controlled comparison, this study aims to elucidate the morphology–structure–performance relationship of γ-alumina adsorbents for fluoride removal, providing new scientific insights into morphology-dependent adsorption behavior and guidance for the rational design of high-performance regenerable alumina adsorbents.

## 2. Results and Discussion

### 2.1. Material, Structural, and Morphological Characterization

**Figure 1:**
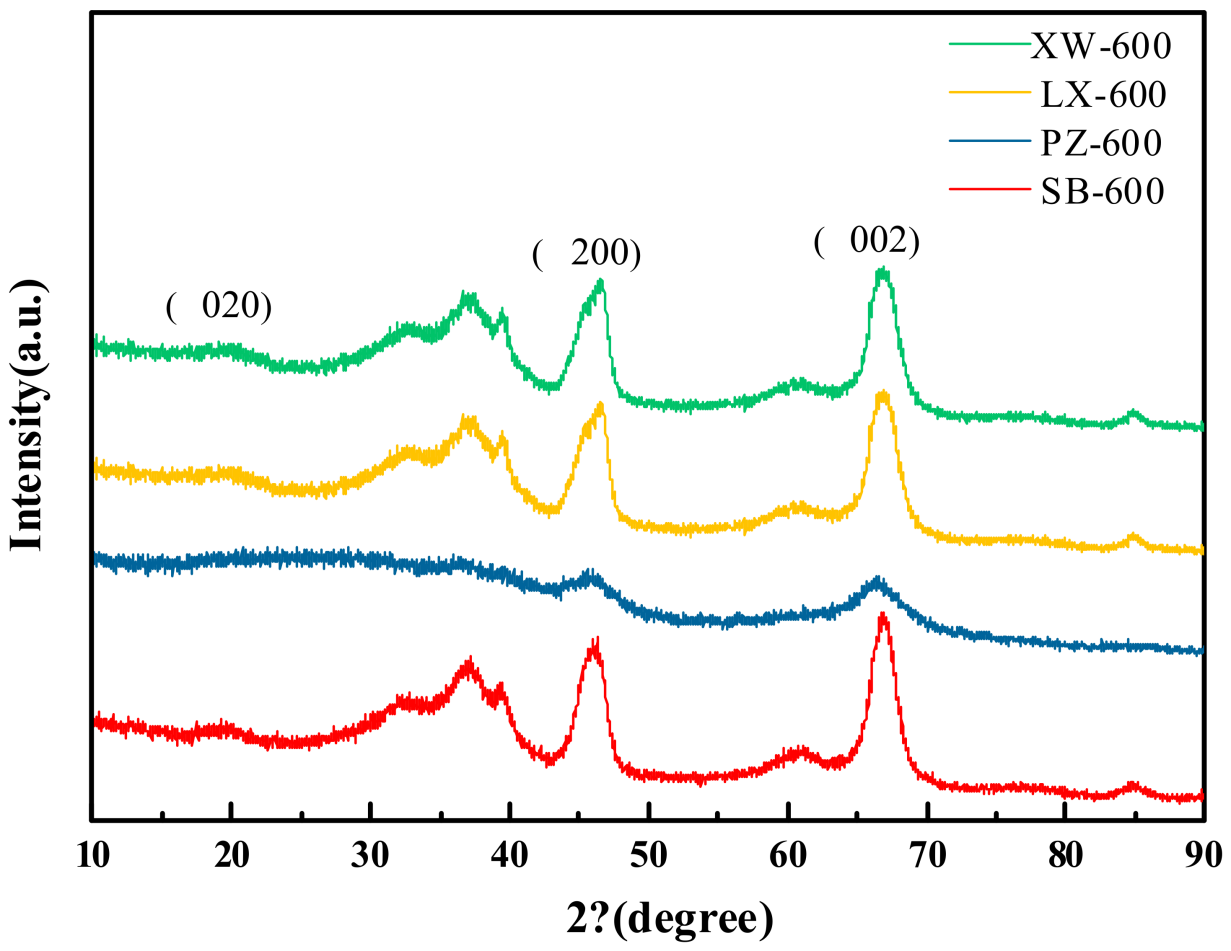
XRD patterns of four alumina samples.

As shown in Figure 1, all four samples only exhibited characteristic diffraction peaks of pure γ-alumina at 2θ = 37.6°, 39.5°, 45.8°, and 66.8°, without any impurity signals, which verified the complete phase transition of boehmite into γ-alumina after calcination. The disappearance of the (020) diffraction peak of sheet PZ-600 indicated an extremely small crystallite size along the b-axis of sheet nanocrystals.

**Figure 2:**
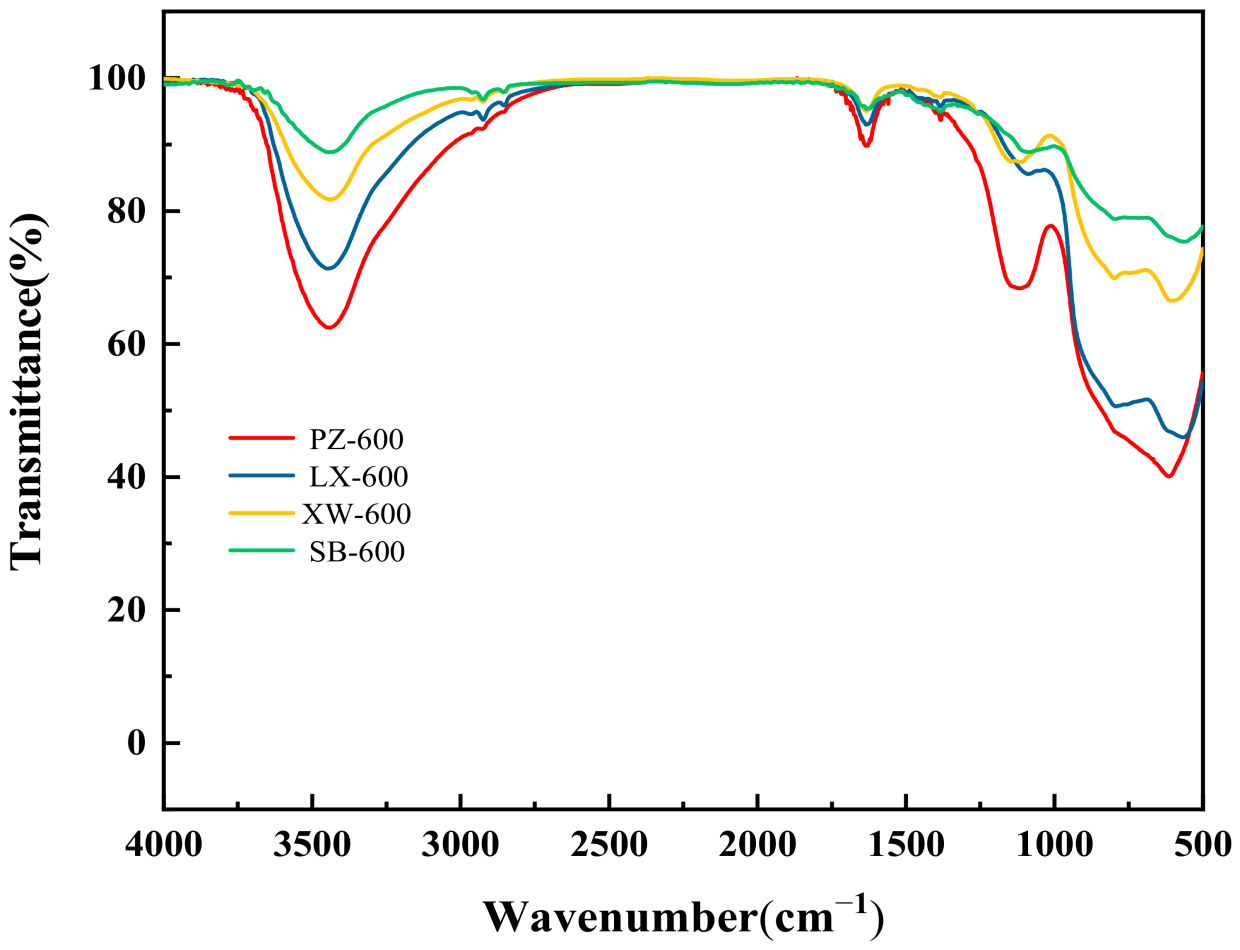
FTIR spectra of alumina samples with different micromorphologies.

FTIR spectra of four alumina samples are shown in Figure 2. A broad absorption band between 500 and 1000 cm^−1^ corresponds to Al–O stretching vibrations of the alumina lattice. The peak centered at 1643 cm^−1^ is assigned to the bending vibration of adsorbed water molecules, while the intense broad band near 3455 cm^−1^ originates from surface hydroxyl stretching. The hydroxyl absorption bands of PZ-600, LX-600, and XW-600 are significantly broader and stronger than SB-600, indicating a higher total hydroxyl content. The sequence of total surface hydroxyl abundance follows PZ-600 > LX-600 > XW-600 > SB-600, demonstrating that sheet alumina possessed the highest density of surface hydroxyl active sites.

**Figure 3:**
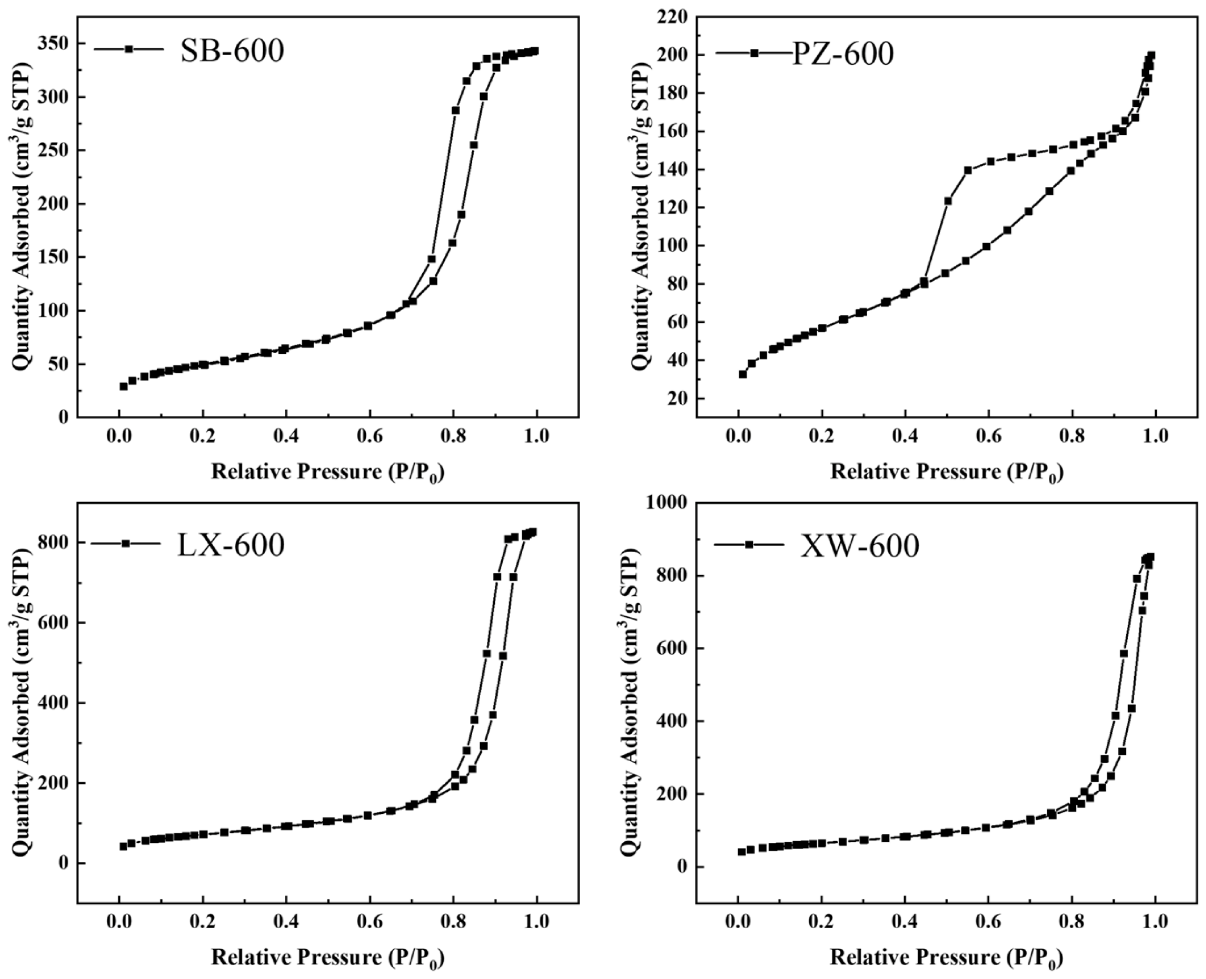
N_2_ adsorption–desorption isotherm curves of alumina samples.

**Figure 4:**
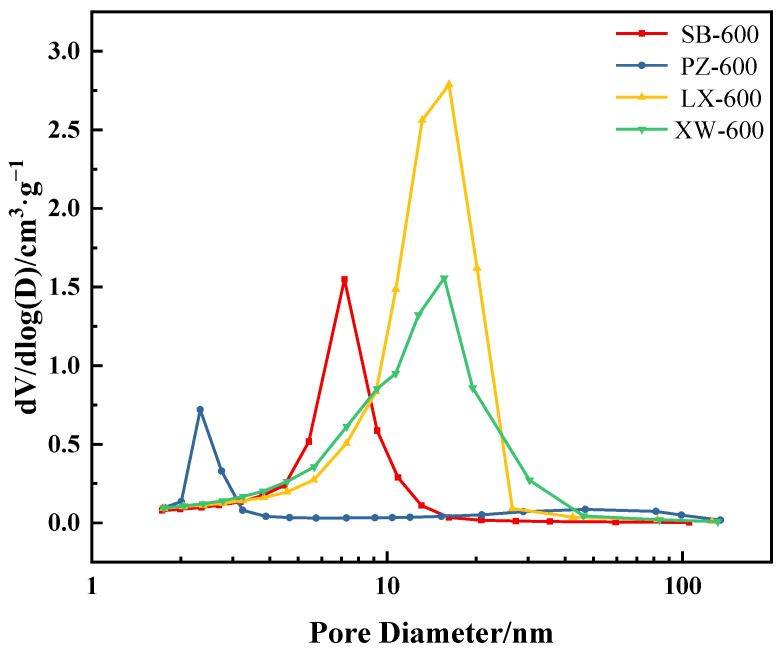
Pore diameter distribution of XW-600, LX-600, PZ-600, and SB-600.

Figure 3 and Figure 4 illustrate low-temperature N_2_ adsorption–desorption isotherms and BJH pore size distributions; Table 1 summarizes textural parameters. SB-600 and LX-600 show Type IV isotherms with H2 hysteresis loops (ink-bottle mesopores). PZ-600 displays Type IV isotherms with H3 loops formed by aggregated nanosheets, consistent with HRTEM observations [27,28]. XW-600 exhibits Type IV isotherms with H4 hysteresis loops corresponding to wedge-shaped slit pores. All samples possess uniform pore size distributions and comparable BET areas (180–230 m^2^∙g^−1^). SB-600 has an average pore diameter of 7.2 nm with a broad pore range of 2–28 nm; PZ-600 features narrow pores centered at 2.3 nm; LX-600 and XW-600 share larger average pore sizes (~16 nm).

**Figure 5:**
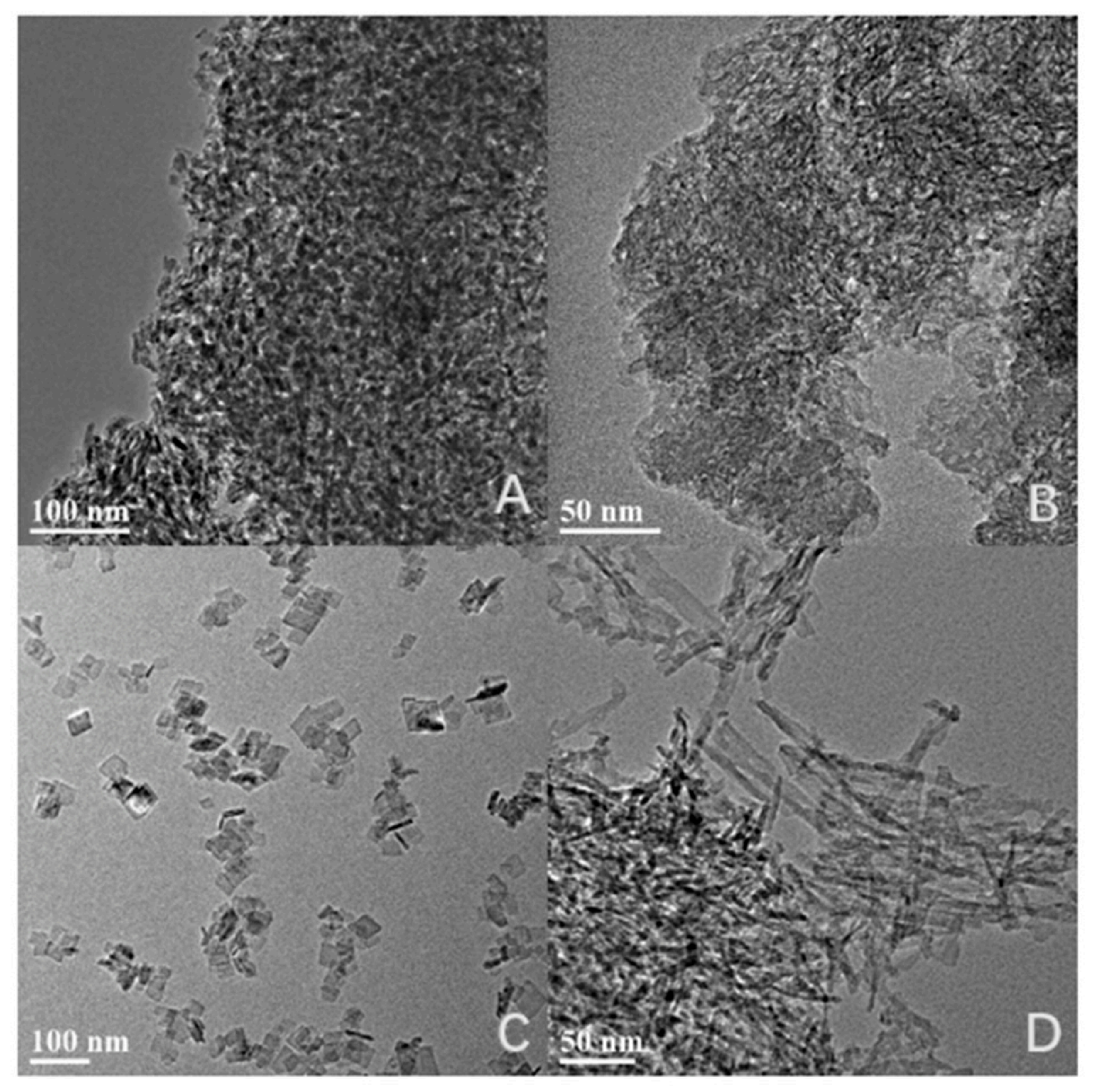
HRTEM images of alumina samples: (**A**) SB-600, (**B**) PZ-600, (**C**) LX-600, (**D**) XW-600.

Figure 5 presents HRTEM images of four alumina samples. HRTEM observations confirmed that the calcined γ-alumina completely inherited the primary micromorphology of corresponding boehmite precursors: SB-600 consisted of spherical nanoparticles; PZ-600 was composed of stacked ultrathin nanosheets; LX-600 displayed regular rhombic flakes; XW-600 showed linear fibrous nanostructures.

**Figure 6:**
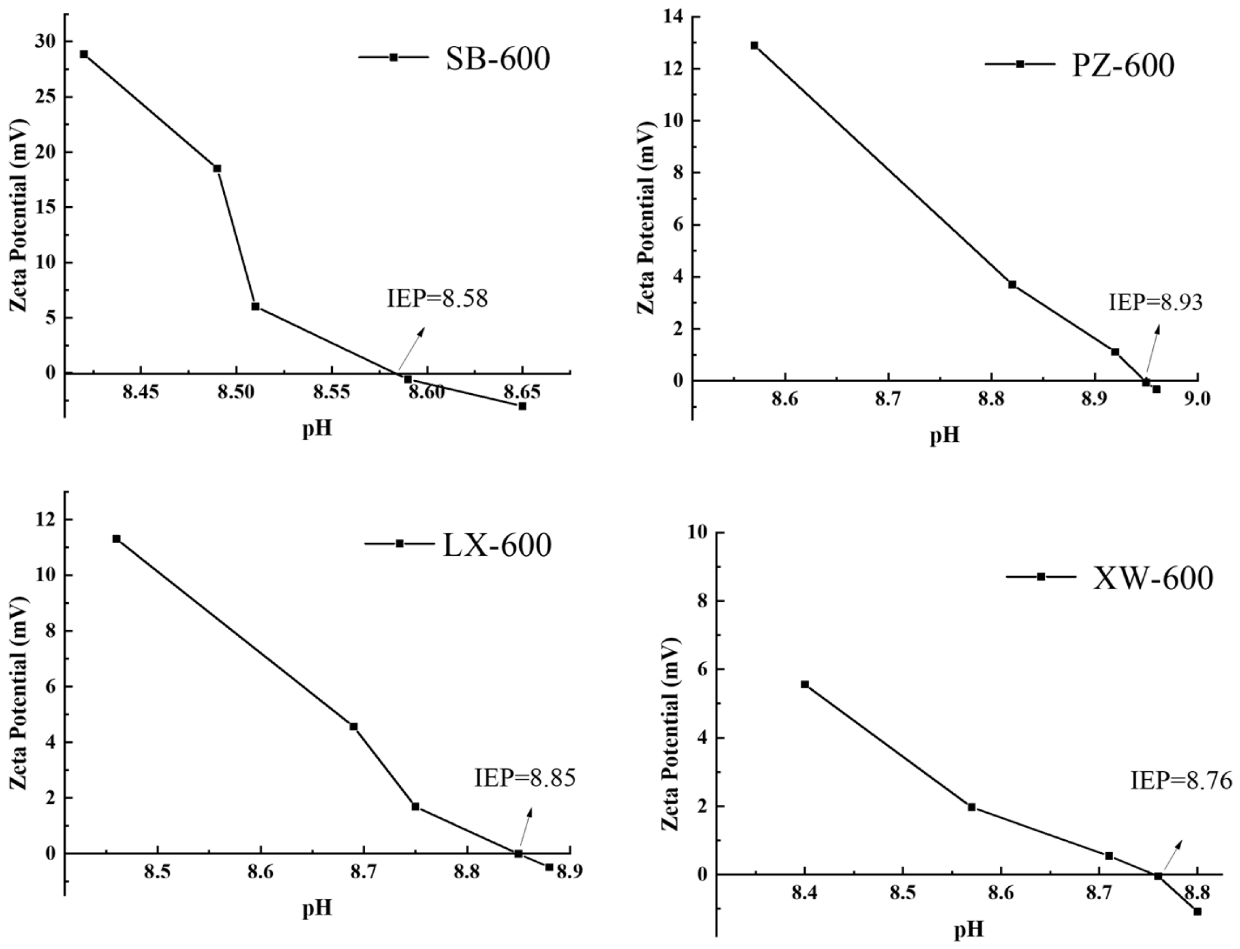
Zeta potential-pH curves of alumina adsorbents.

As revealed in Figure 6, the isoelectric points (IEPs) followed the sequence PZ-600 (8.93) > LX-600 (8.85) > XW-600 (8.76) > SB-600 (8.58), consistent with the order of total surface hydroxyl content. γ-Alumina is a typical amphoteric oxide, and the density of surface hydroxyl groups varies with solution pH [25,26]. In practical wastewater treatment, the solution pH is generally lower than the IEP of alumina. Under such circumstances, alumina surfaces are positively charged and generate strong electrostatic attraction toward fluoride anions, endowing alumina with outstanding aqueous defluoridation capacity.

### 2.2. Aqueous Fluoride Adsorption Performance

#### 2.2.1. Adsorption Kinetics

**Figure 7:**
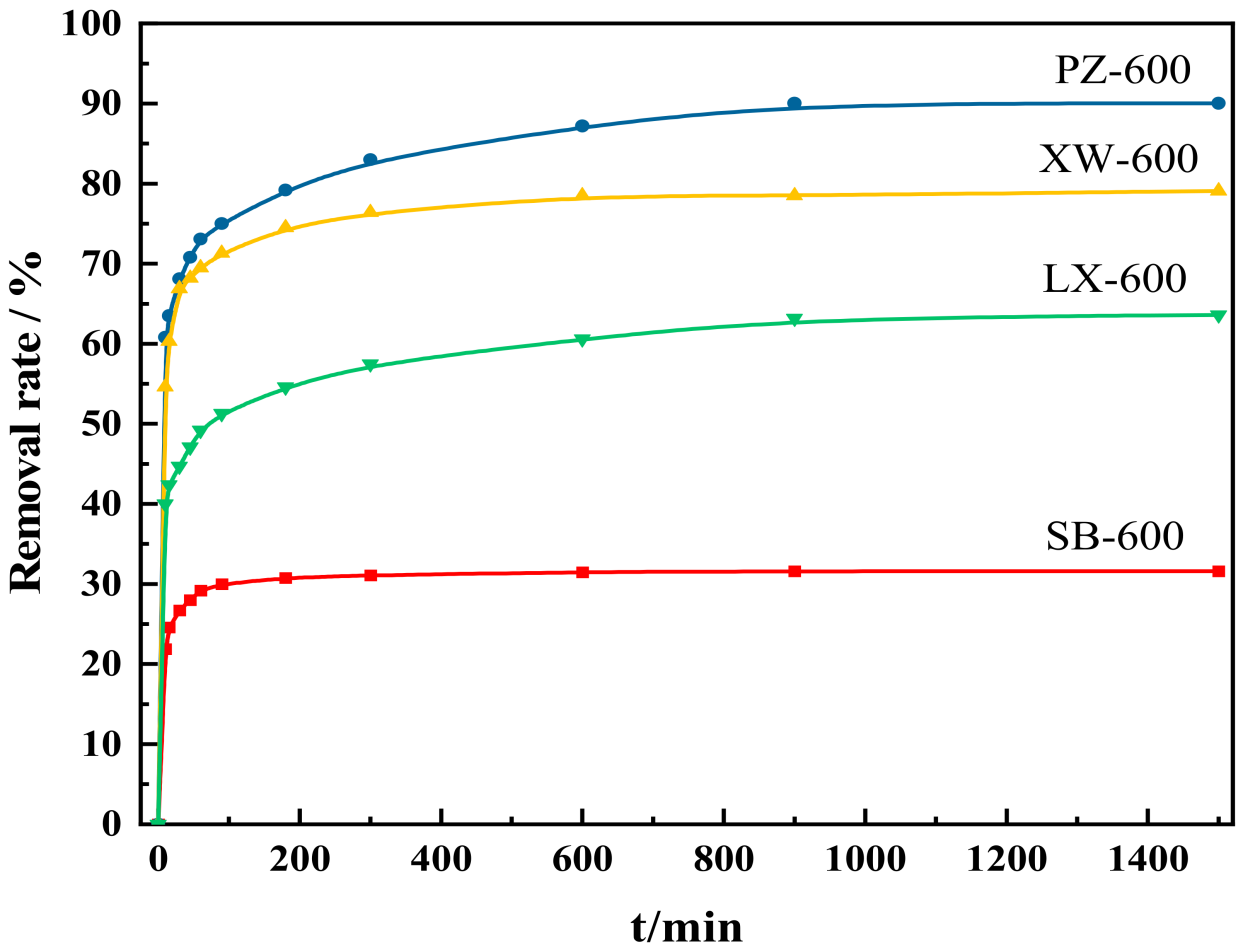
Time-dependent fluoride adsorption capacity curves of alumina samples.

Time-dependent fluoride uptake curves are presented in Figure 7. Commercial SB-600 reached adsorption equilibrium within approximately 300 min, whereas PZ-600, LX-600, and XW-600 required more than 1440 min to approach equilibrium. Compared with commercial alumina, the laboratory-synthesized samples exhibited slower adsorption kinetics. This behavior is likely associated with the combined effects of diffusion limitations and the larger number of available adsorption sites. The stacked nanosheet and rhombic flake architectures may create more tortuous diffusion pathways, increasing the mass transfer resistance of fluoride ions. In addition, the higher adsorption capacities of the synthesized alumina samples require a longer time for fluoride ions to occupy the available active sites before equilibrium is reached.

All samples exhibited comparable initial adsorption rates during the first 100 min, which may be attributed to the high concentration gradient and the abundance of freely available fluoride ions at the beginning of the adsorption process. The equilibrium fluoride removal efficiencies followed the order PZ-600 (90.05%) > XW-600 > LX-600 > SB-600 (31.6%). Although PZ-600 possessed a slightly lower BET surface area than LX-600, it exhibited the highest equilibrium adsorption capacity, suggesting that specific surface area alone is insufficient to explain the adsorption behavior. Likewise, the higher fluoride adsorption capacity of XW-600 compared with LX-600, despite their comparable surface hydroxyl contents, indicates that the accessibility and local chemical environment of surface hydroxyl species may also influence fluoride adsorption. This interpretation is consistent with previous studies suggesting that hydroxyl groups located at edge or defect sites generally exhibit higher reactivity than those on terrace planes [31,32,33,34], although the present work does not directly identify or distinguish these hydroxyl species. Kinetic fitting results (Table 2) showed that the pseudo-second-order model provided excellent correlations (R^2^ > 0.998) for all four materials.

#### 2.2.2. Adsorption Isotherms

**Figure 8:**
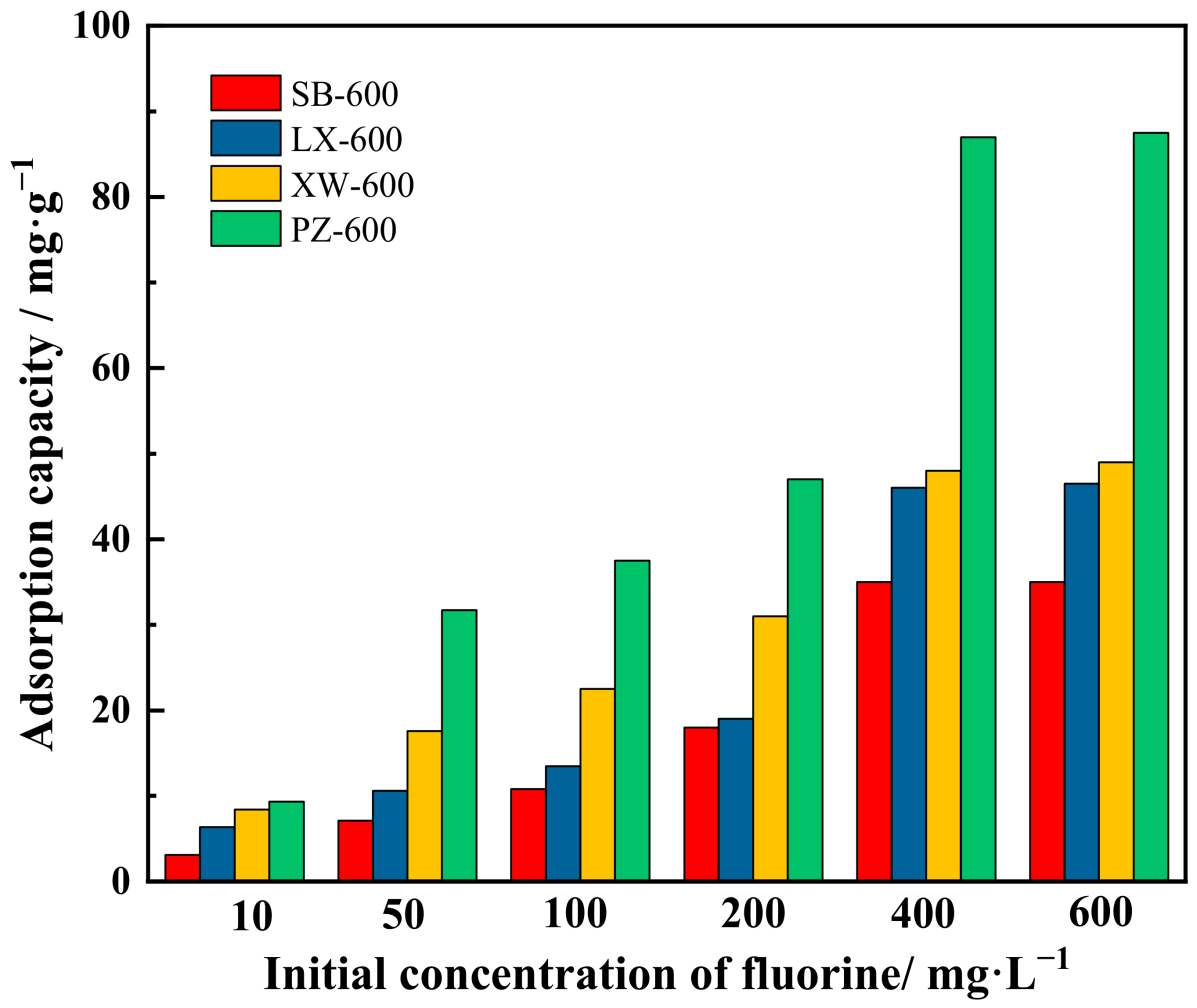
Effect of initial fluoride concentration on the adsorption capacity of alumina.

Equilibrium adsorption capacities at initial fluoride concentrations ranging from 10 to 600 mg·L^−1^ are presented in Figure 8. The adsorption capacities increased progressively with increasing initial fluoride concentration and gradually approached a plateau above 400 mg·L^−1^, suggesting that the available adsorption sites became nearly saturated at high fluoride loadings. FTIR analysis indicated that LX-600 possessed a higher overall surface hydroxyl content than XW-600. However, despite this higher hydroxyl density, the adsorption capacities at an initial fluoride concentration of 600 mg·L^−1^ followed the order of PZ-600 (87.2 mg·g^−1^) > XW-600 (49.0 mg·g^−1^) > LX-600 (46.1 mg·g^−1^) > SB-600 (35.0 mg·g^−1^). This result suggests that the total amount of surface hydroxyl groups is not the sole factor governing fluoride adsorption. Instead, differences in the accessibility, local chemical environment, and reactivity of surface hydroxyl species associated with different alumina morphologies are likely to influence adsorption performance. Among the four samples, PZ-600 exhibited the largest increase in fluoride uptake with increasing fluoride concentration, suggesting that its nanosheet morphology provides a greater number of accessible adsorption sites or more favorable adsorption environments under high fluoride loading.

The effects of solution pH and coexisting ions on fluoride adsorption were further evaluated, and the corresponding results have been included in the Supplementary Materials (Figures S1 and S2). The effects of representative coexisting anions (PO_4_^3−^, NO_3_^−^, Cl^−^, and SO_4_^2−^) are presented in Figure S2. Among these anions, PO_4_^3−^ exhibited the strongest inhibitory effect on fluoride adsorption because of its stronger affinity for surface hydroxyl sites, resulting in pronounced competitive adsorption. In contrast, NO_3_^−^, Cl^−^, and SO_4_^2−^ had only a minor influence on the laboratory-synthesized alumina samples, whereas commercial SB-600 was more susceptible to interference from these coexisting anions. These results indicate that the morphology-controlled alumina adsorbents exhibit good fluoride selectivity in the presence of common coexisting anions, while phosphate is the primary competitive species.

Langmuir and Freundlich isotherm models were adopted to fit the equilibrium data to quantify the correlation between equilibrium adsorption capacity and residual fluoride concentration; the fitting parameters are listed in Table 3. All four alumina samples were better described by the Freundlich isotherm model (R^2^ > 0.996), indicating that fluoride adsorption occurred on energetically heterogeneous surfaces with a non-uniform distribution of adsorption sites and adsorption energies. The Freundlich isotherm is an empirical model widely used to describe adsorption on heterogeneous surfaces rather than to identify specific adsorption mechanisms [35,36]. The 1/n values of the three laboratory-synthesized alumina samples were all below 0.5, indicating favorable fluoride adsorption. In contrast, the Langmuir model provided relatively poor fits for SB-600 (R^2^ = 0.6758) and XW-600 (R^2^ = 0.5345), suggesting that the assumption of a homogeneous surface with energetically equivalent adsorption sites was not fully applicable to these materials. The relatively poor Langmuir fit for XW-600 may be associated with its nanofibrous architecture, which is likely to create adsorption sites with different local chemical environments and adsorption affinities. This interpretation is further supported by the relatively low 1/n value (0.2954), indicating a higher degree of surface heterogeneity. Nevertheless, the present results do not directly identify the nature or distribution of specific adsorption sites, and this interpretation should therefore be regarded as a reasonable hypothesis based on the adsorption behavior together with previous studies.

#### 2.2.3. Adsorption Mechanism via XPS Analysis

**Figure 9:**
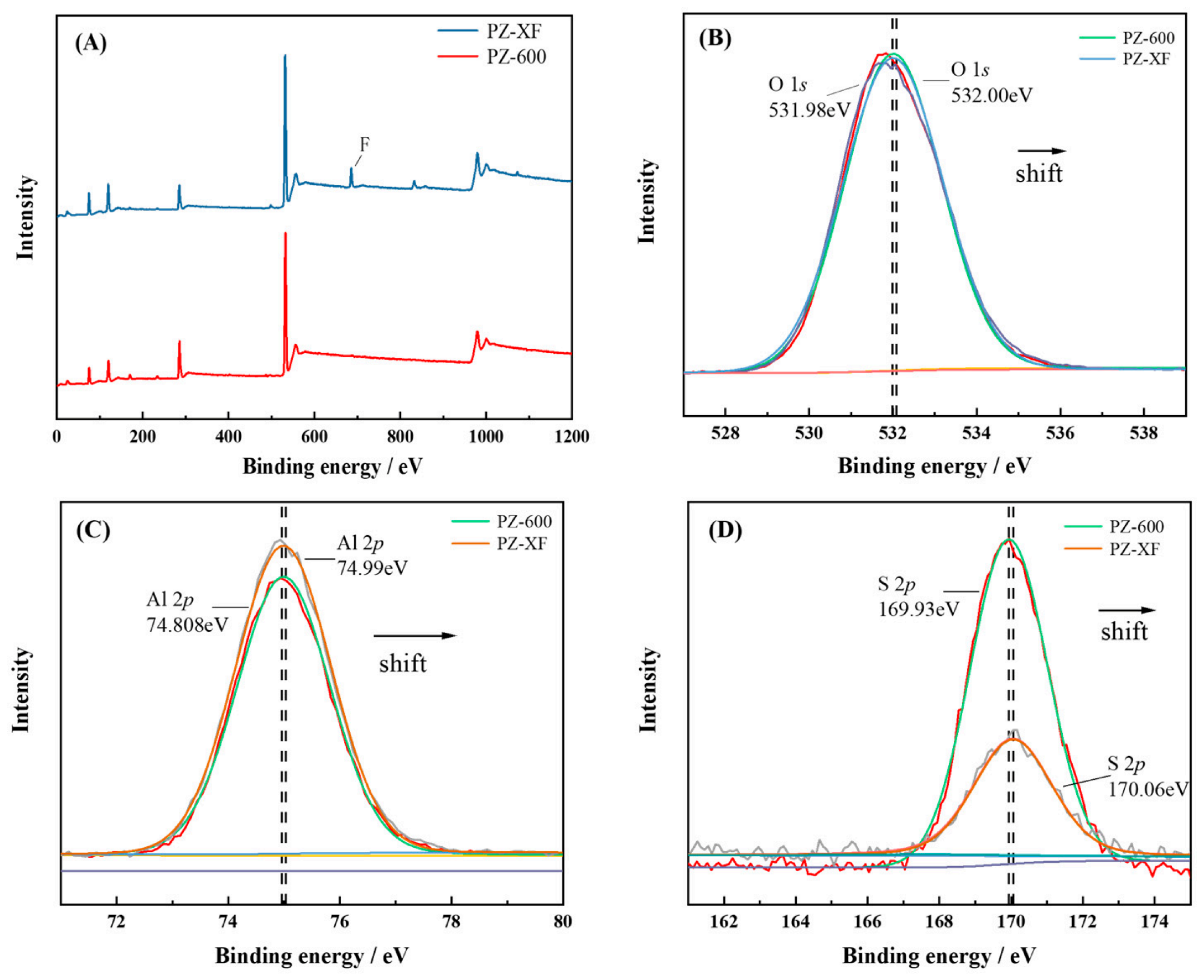
XPS full spectrum and high-resolution spectra of PZ-600 before and after fluoride adsorption: (**A**) full scan, (**B**) O 1s, (**C**) Al 2p, (**D**) S 2p.

**Figure 10:**
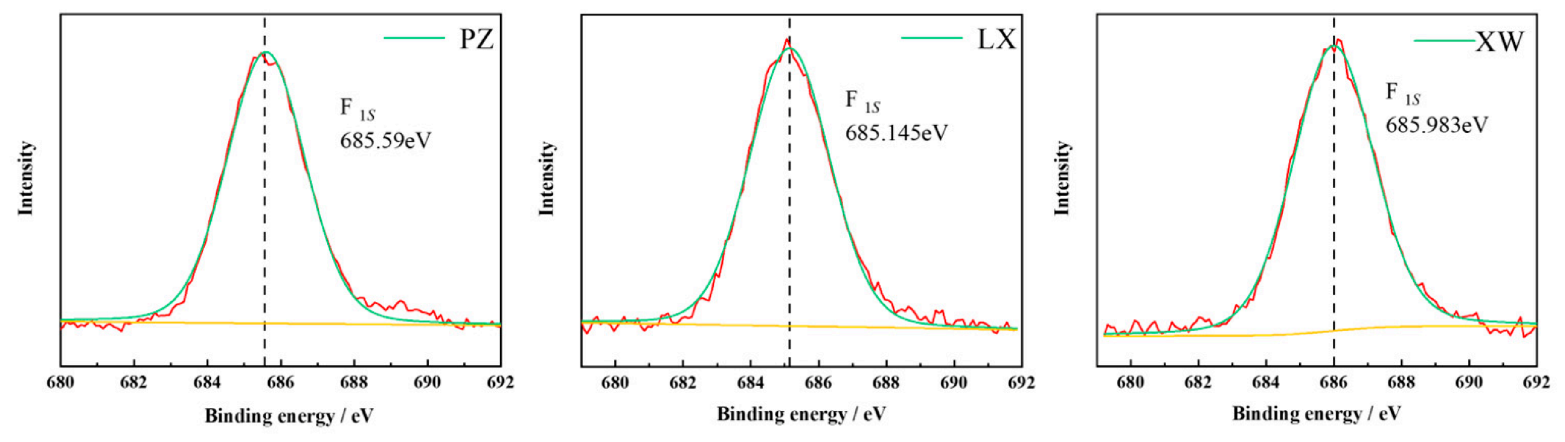
High-resolution F 1s XPS spectra of PZ-600, LX-600, and XW-600 after fluoride adsorption.

Previous studies have identified ligand exchange between surface Al–OH groups and aqueous fluoride ions to form Al–F species as the dominant defluoridation pathway [15]. The XPS survey and high-resolution spectra of PZ-600 before and after fluoride adsorption are presented in Figure 9. After fluoride adsorption, a distinct F 1s signal appears on the saturated sample, indicating successful fluoride uptake. Figure 10 shows the F 1s spectra of the three laboratory-synthesized alumina samples after fluoride adsorption. The F 1s binding energies fall within the range of 685–686 eV, which is consistent with the reported binding energy of Al–F bonds in AlF_3_·3H_2_O [37,38,39,40]. These results are consistent with the formation of Al–F species during fluoride adsorption. In addition, the Al 2p and O 1s spectra exhibit discernible shifts toward higher binding energies after fluoride adsorption, indicating changes in the chemical environment of surface aluminum and oxygen species. The weak S 2p signal originates from trace sulfate species remaining after synthesis using aluminum sulfate as the precursor. Since sulfur is not involved in fluoride adsorption, the S 2p spectra are not considered in the mechanistic discussion. Overall, the XPS results suggest the involvement of surface hydroxyl groups in the adsorption process through ligand exchange with fluoride ions. However, the present XPS analysis is primarily qualitative, and therefore the proposed adsorption mechanism should be regarded as a plausible interpretation supported by indirect evidence rather than definitive proof. The plausible interfacial reaction pathways are summarized as follows [15,34]:

Al–OH_(s)_ + F^−^_(l)_ → Al–F_(s)_ + OH^−^_(aq)_

Al–OH_2_^+^ + F^−^_(l)_ → Al–F_(s)_ + H_2_O

### 2.3. Cyclic Regeneration Stability Evaluation

#### 2.3.1. Material, Structural, and Morphological Characterization After Regeneration

Adsorbent regeneration reduces operational costs and secondary pollution for practical water treatment [34]. A two-step regeneration strategy (NaOH desorption followed by dilute HCl reactivation) was adopted to evaluate cyclic performance, and multi-characterization methods were applied to track structural variations after repeated acid–base treatment.

**Figure 11:**
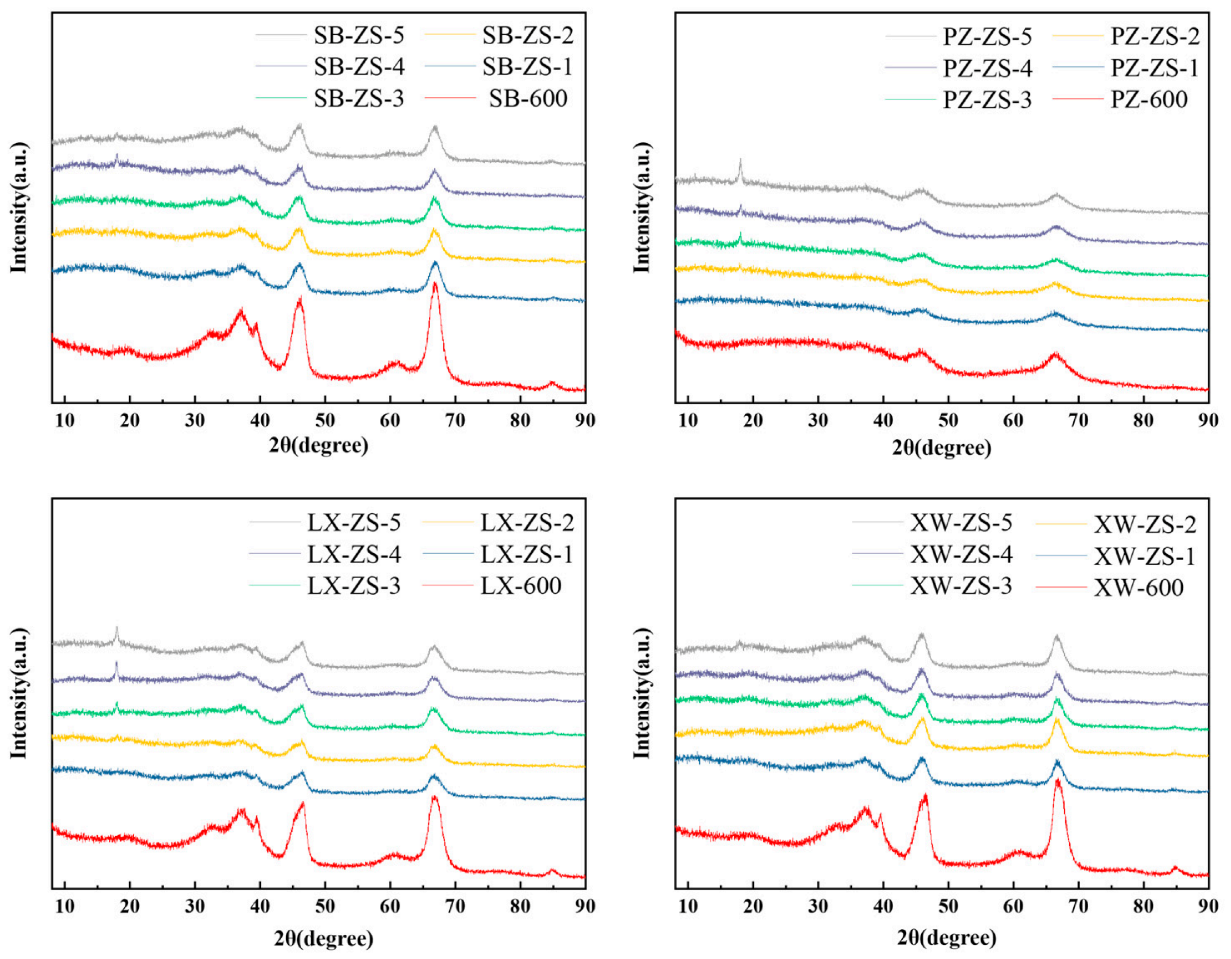
XRD patterns of alumina before and after regeneration cycles.

Figure 11 displays XRD patterns of alumina before and after five adsorption–desorption cycles. All samples retained the main characteristic peaks of γ-alumina, yet the peak half-widths broadened obviously, indicating decreased crystallinity after repeated acid-base treatment. PZ-600 and LX-600 generated Al (OH)_3_ impurity peaks after two regeneration runs; SB-600 formed aluminum hydroxide precipitates after three cycles, whereas fibrous XW-600 only presented weak Al (OH)_3_ signals after five cycles, demonstrating superior acid-base structural stability of fibrous nanostructures.

**Figure 12:**
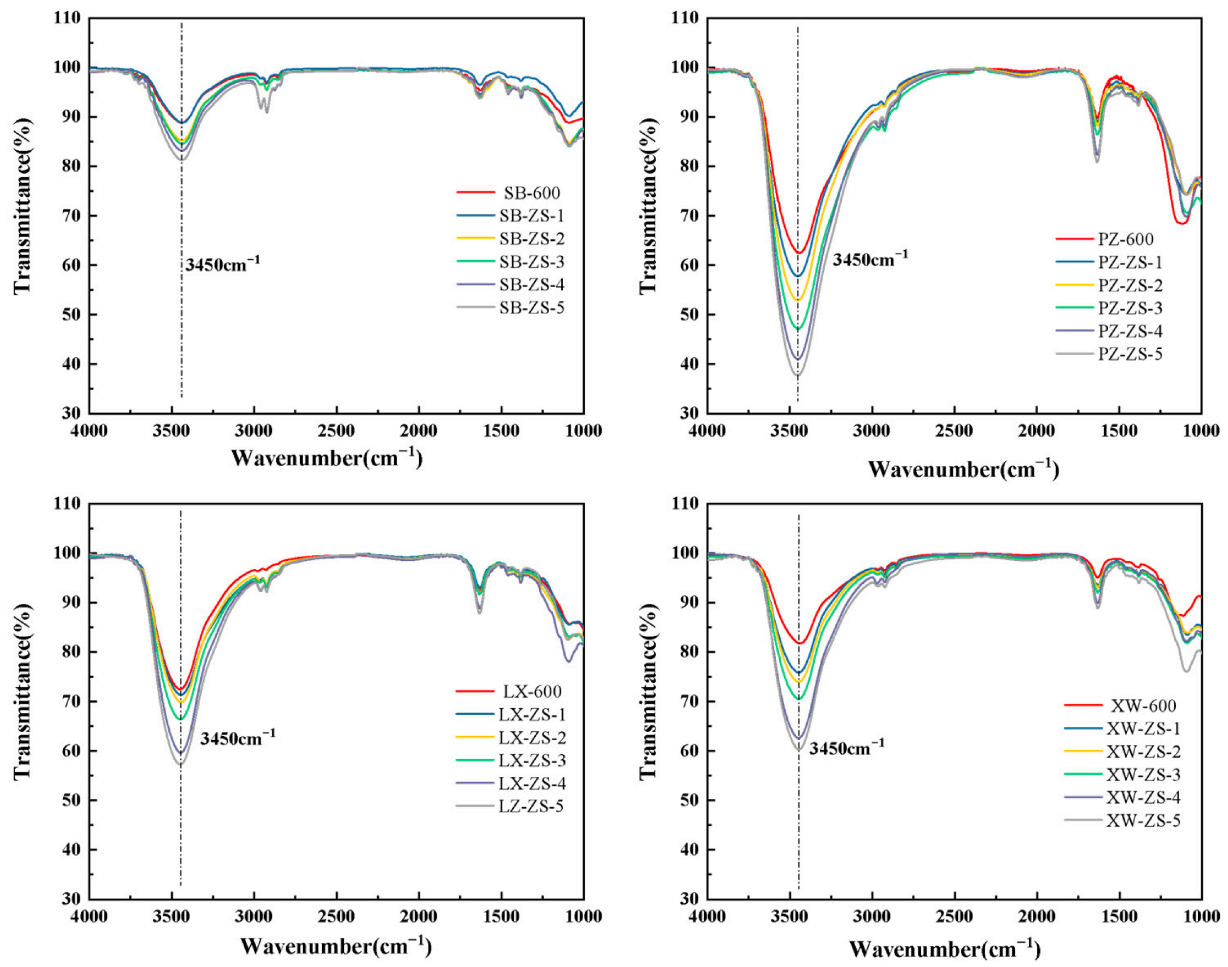
FTIR spectra of alumina samples before and after regeneration cycles.

As illustrated in Figure 12, the intensity of the O-H stretching band at 3450 cm^−1^ gradually increased with more regeneration cycles, which could be attributed to two factors: partial hydrolysis of alumina generating aluminum hydroxide and residual bound water trapped on material surfaces.

**Figure 13:**
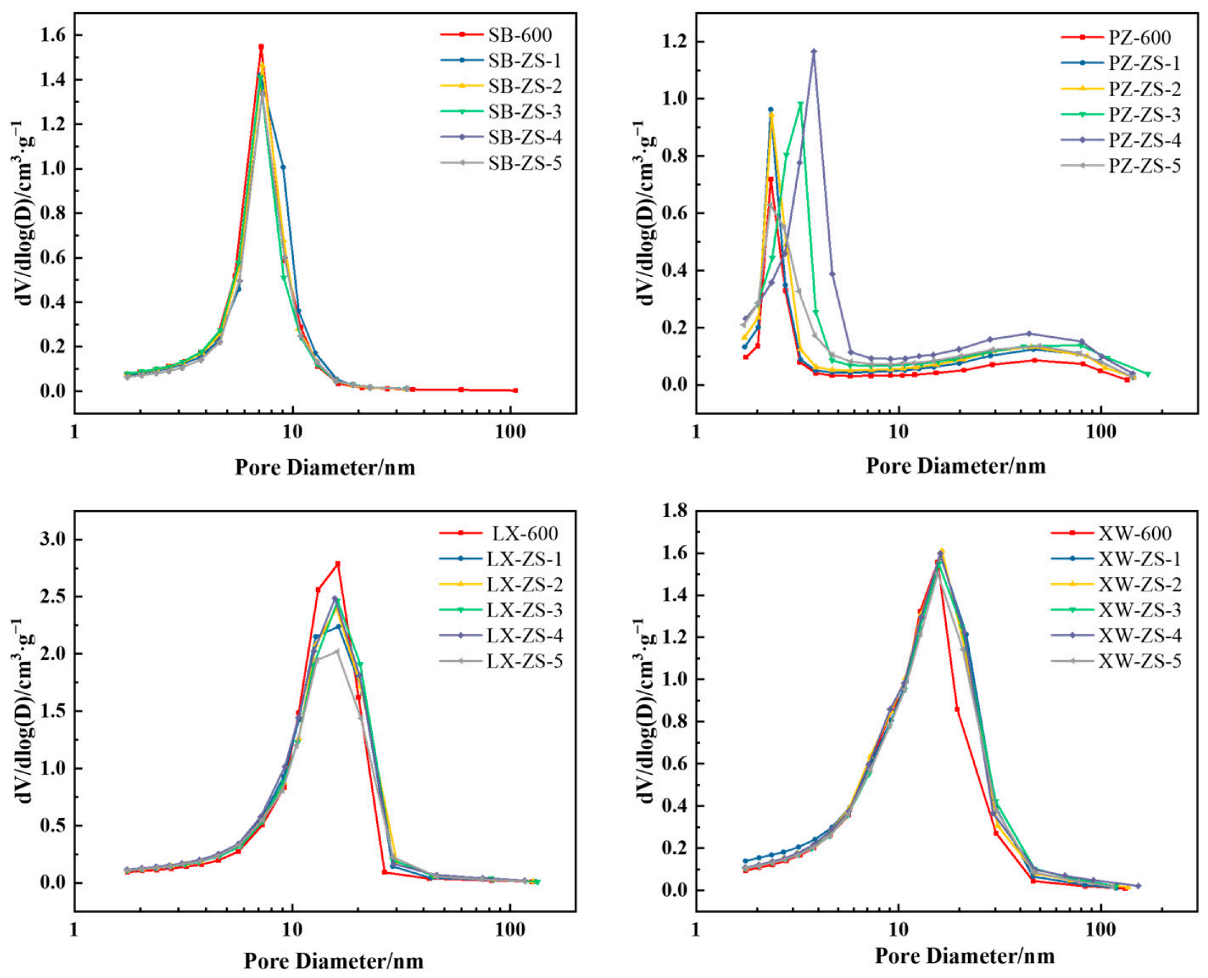
Pore size distribution curves of alumina before and after regeneration cycles.

Combining Figure 13 and Table 4, SB-600 exhibited continuous declines in specific surface area and pore volume with cycling. The BET parameters of PZ-600 presented a dramatic rise-and-fall trend: the remarkable increase in BET surface area is likely associated with an alkaline dissolution–reprecipitation process rather than simple surface etching. During regeneration, slight dissolution of the alumina framework under alkaline conditions, reopening of previously inaccessible pores, removal of fluoride-containing surface deposits, and partial reconstruction of the pore network collectively increase the accessible surface area and pore volume. With repeated regeneration, however, hydrolysis and reprecipitation of aluminum species may produce Al (OH)_3_ deposits that partially block mesopores, leading to the reduction in pore volume and average pore diameter observed after the fifth regeneration cycle. In contrast, LX-600 and XW-600 maintained nearly unchanged specific surface area, pore volume, and average pore size over five cycles, demonstrating robust pore structural stability.

**Figure 14:**
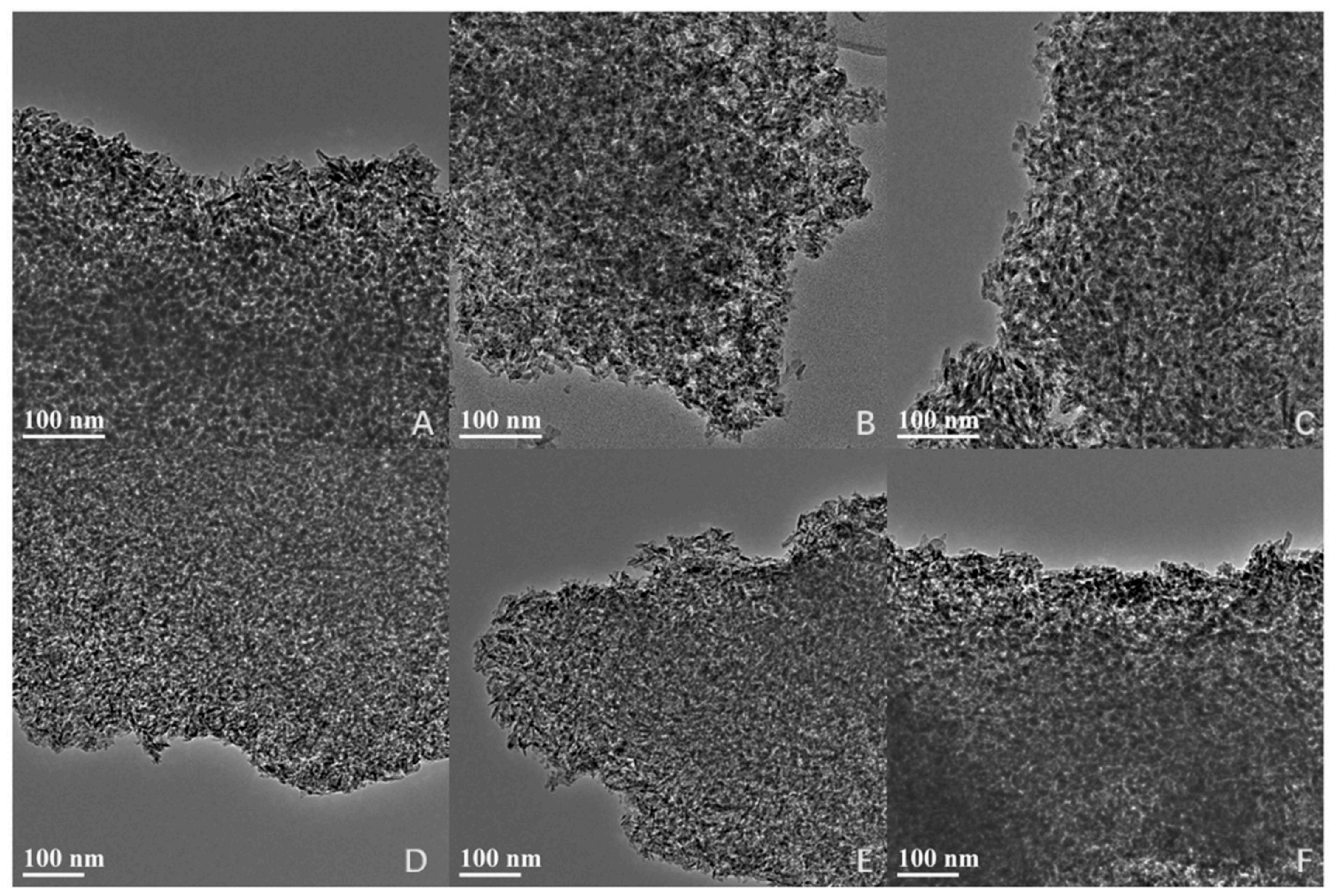
HRTEM images of SB-600 over regeneration cycles: (**A**) raw, (**B**) ZS-1, (**C**) ZS-2, (**D**) ZS-3, (**E**) ZS-4, (**F**) ZS-5.

**Figure 15:**
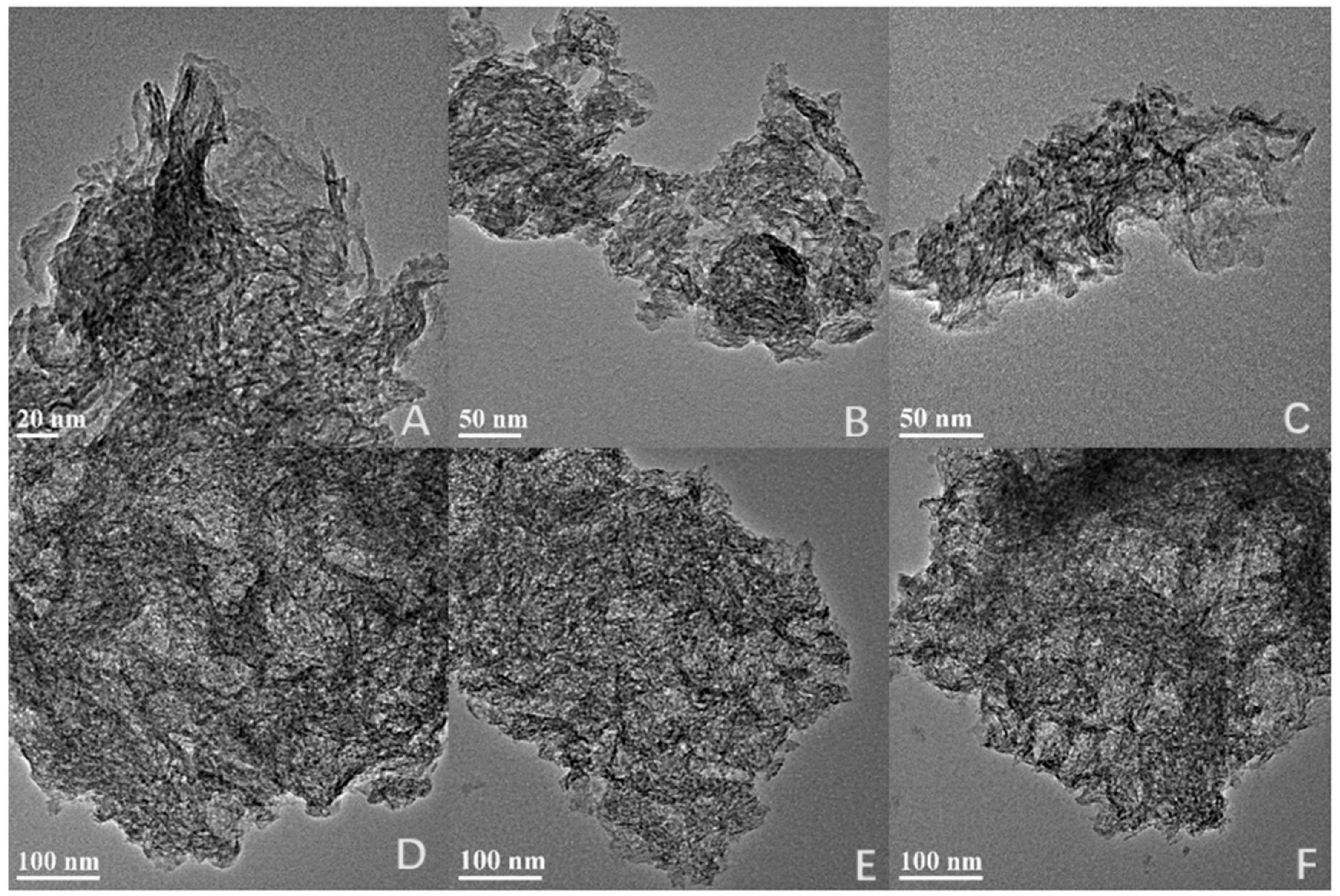
HRTEM images of PZ-600 over regeneration cycles: (**A**) raw, (**B**) ZS-1, (**C**) ZS-2, (**D**) ZS-3, (**E**) ZS-4, (**F**) ZS-5.

**Figure 16:**
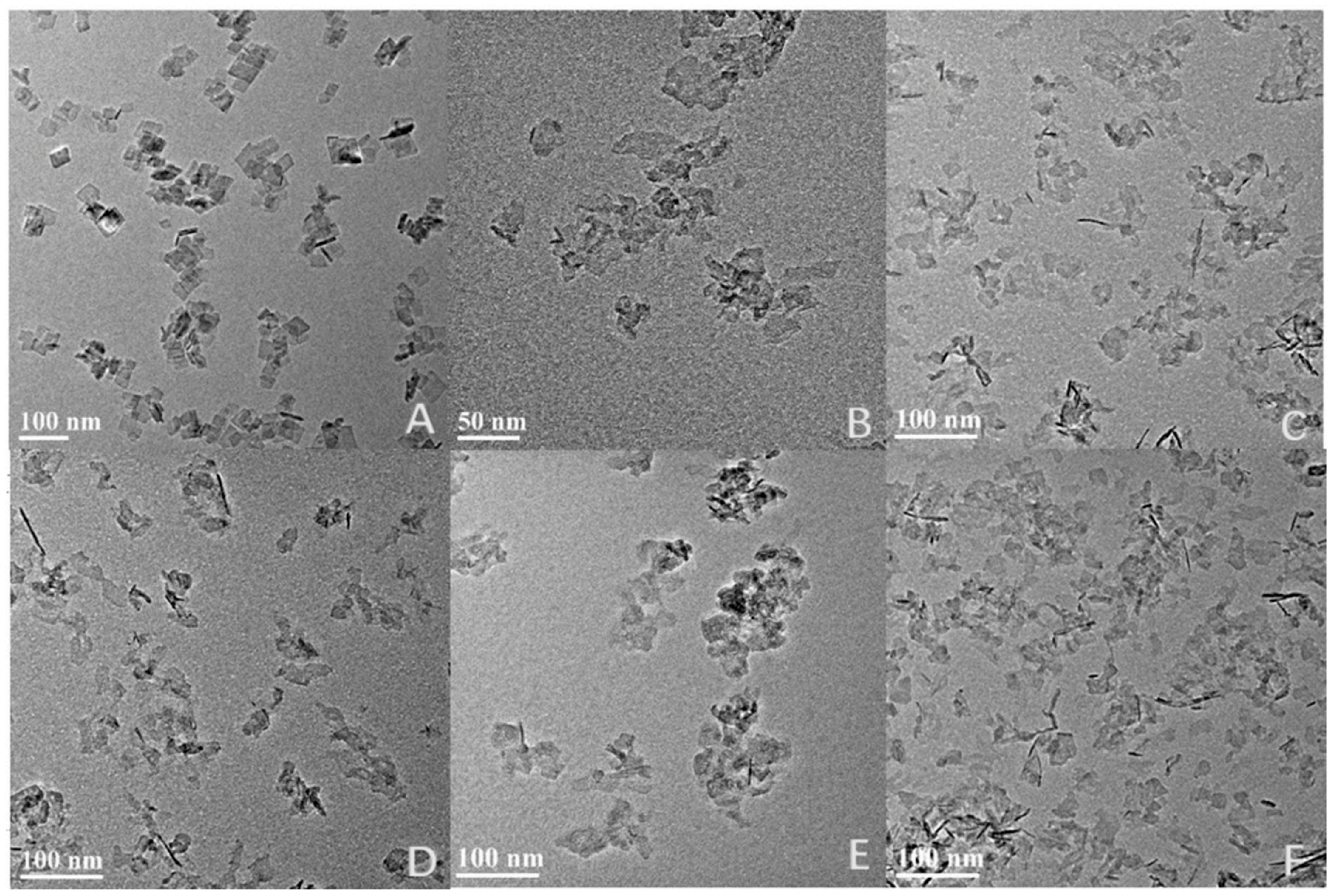
HRTEM images of LX-600 over regeneration cycles: (**A**) raw, (**B**) ZS-1, (**C**) ZS-2, (**D**) ZS-3, (**E**) ZS-4, (**F**) ZS-5.

**Figure 17:**
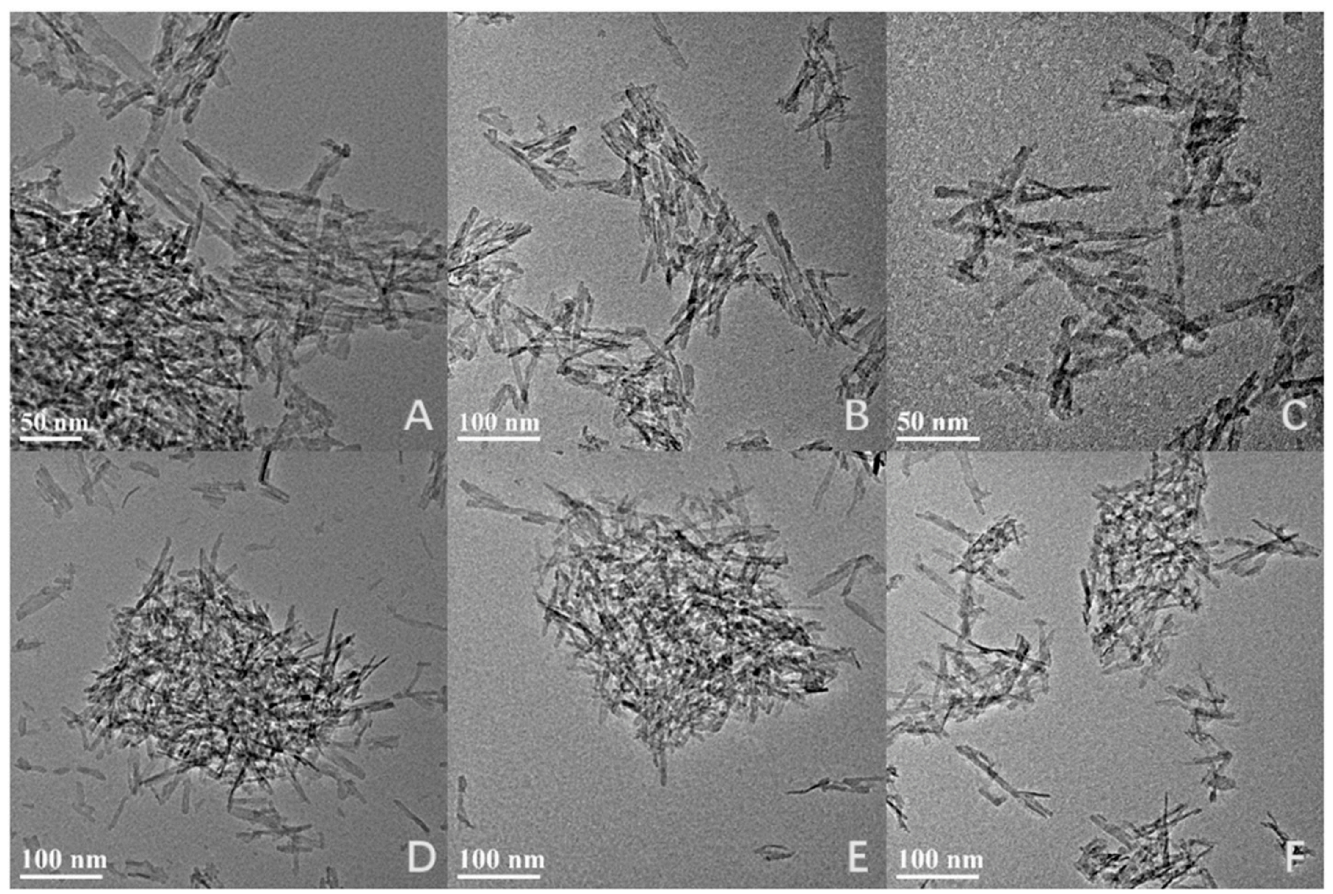
HRTEM images of XW-600 over regeneration cycles: (**A**) raw, (**B**) ZS-1, (**C**) ZS-2, (**D**) ZS-3, (**E**) ZS-4, (**F**) ZS-5.

HRTEM images (Figure 14, Figure 15, Figure 16 and Figure 17) revealed obvious morphological degradation after five cycles. Spherical SB-600 and sheet PZ-600 suffered severe particle agglomeration with drastically reduced inter-particle voids; rhombic LX-600 lost its regular flaky geometry and turned irregular. By contrast, fibrous XW-600 retained complete linear nanostructures without apparent aggregation, further verifying its superior acid-base corrosion resistance.

#### 2.3.2. Attenuation of Adsorption Capacity During Cycling

**Figure 18:**
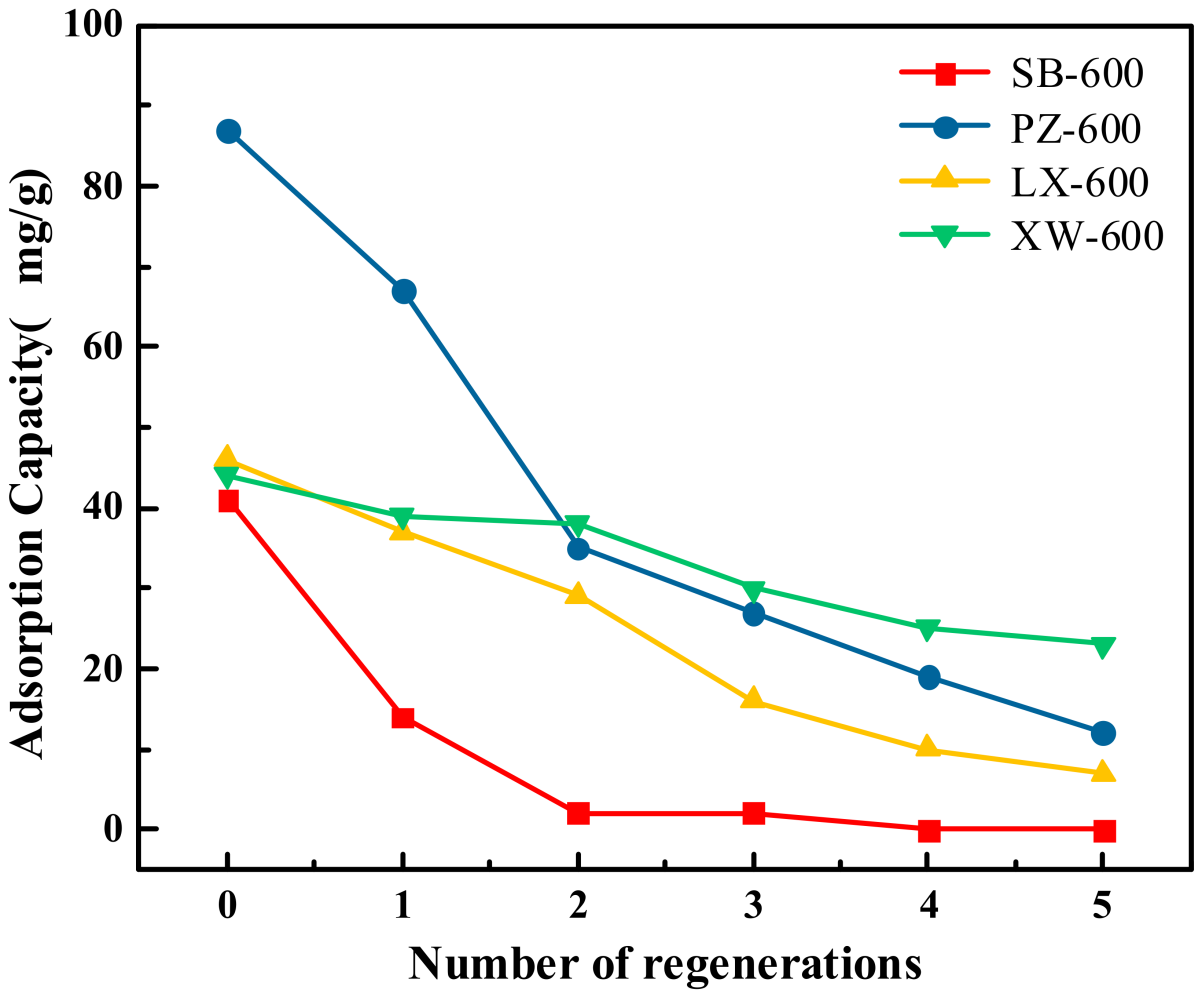
Variations in the adsorption capacity of alumina during five regeneration cycles.

The evolution of fluoride uptake capacity over five regeneration cycles is presented in Figure 18. Commercial SB-600 nearly lost its defluoridation activity after three regeneration cycles. Although PZ-600 exhibited the highest initial adsorption capacity, its performance declined markedly, retaining only 12.4 mg·g^−1^ after five cycles. Similarly, LX-600 retained only 7.1 mg·g^−1^ after the fifth cycle, whereas XW-600 maintained the highest residual adsorption capacity (23.1 mg·g^−1^), demonstrating superior cyclic stability among the synthesized alumina samples. Although the total hydroxyl content increased after regeneration, the newly formed hydroxyl species may possess lower adsorption activity than the original surface hydroxyls. In addition, a fraction of the surface-bound fluoride species may not be completely desorbed during the regeneration process, leading to the progressive loss of effective adsorption sites and a gradual decline in fluoride uptake. The superior regeneration stability of XW-600 is likely associated with its interconnected nanofibrous framework, which provides greater structural integrity and distributes mechanical and chemical stresses more uniformly during repeated acid–alkali treatments. In contrast, the stacked nanosheet architecture of PZ-600 is more susceptible to partial dissolution, structural rearrangement, and layer fragmentation during cyclic regeneration, resulting in a more pronounced deterioration of its adsorption performance. These observations are consistent with previous studies showing that one-dimensional fibrous oxide frameworks generally exhibit greater structural robustness than two-dimensional nanosheet assemblies under repeated chemical treatments [41,42,43].

To further evaluate the significance of the present work, the adsorption performance of the synthesized γ-alumina samples was compared with representative alumina-based fluoride adsorbents reported in the literature (Table 5). Although several previously reported alumina materials exhibit higher specific surface areas or comparable fluoride adsorption capacities, most studies focus primarily on improving adsorption performance through surface area enhancement, compositional modification, or template-assisted synthesis. In contrast, the present work systematically investigates the intrinsic influence of alumina morphology on fluoride adsorption and regeneration by employing γ-alumina samples with comparable BET surface areas. This experimental design minimizes the contribution of specific surface area and provides clearer insight into the morphology–adsorption relationship. Moreover, the template-free hydrothermal synthesis and the superior regeneration stability of the nanofibrous alumina further demonstrate the practical potential of the proposed materials.

## 3. Materials and Methods

### 3.1. Chemicals and Raw Materials

Analytical-grade sodium hydroxide, octadecahydrate aluminum sulfate, sodium fluoride, disodium hydrogen phosphate dodecahydrate, glacial acetic acid, trans-1,2-cyclohexanediaminetetraacetic acid (CDTA), concentrated HCl (36–38wt%), concentrated HNO_3_ (65–68wt%), and potassium nitrate were purchased from Xilong Scientific and Fuchen Chemical Reagent Co., Ltd. (Shantou, China). Commercial boehmite powder (SB series) was supplied by Sasol GmbH (Wuhan, China). All reagents were used as received without further purification.

### 3.2. Synthesis of Adsorbents

Sheet, rhombic sheet, and fibrous boehmite precursors were prepared by hydrothermal neutralization of sodium metaaluminate and aluminum sulfate aqueous solutions. The as-prepared boehmite powders were calcined at 600 °C for 2 h to induce a phase transition to γ-alumina. Four alumina samples with similar BET surface areas were selected for subsequent fluoride adsorption tests and labeled according to their micromorphology and calcination temperature:

PZ-600: sheet-structured γ-alumina calcined at 600 °C;

LX-600: rhombic sheet γ-alumina calcined at 600 °C;

XW-600: fibrous γ-alumina calcined at 600 °C;

SB-600: commercial spherical γ-alumina (Sasol SB precursor calcined at 600 °C).

The detailed hydrothermal synthesis parameters, including hydrothermal temperature, reaction time, and pH conditions for preparing PZ, LX, and XW, are summarized in Table S1 in the Supplementary Materials.

### 3.3. Material Characterizations

X-ray Diffraction (XRD): Bruker D8 ADVANCE powder diffractometer (Bruker AXS, Karlsruhe, Germany) with Cu Kα radiation (graphite monochromator). Diffraction profiles were matched with standard PDF cards to identify crystalline phases. High-Resolution Transmission Electron Microscopy (HRTEM): JEOL JEM-2100 instrument (JEOL Ltd, Tokyo, Japan) to record particle micromorphology at the nanoscale. N_2_ Adsorption–Desorption Isotherms: Micromeritics TriStar 3020 analyzer (Micromeritics Instrument Corporation, Norcross, GA, USA) operated at 77 K to determine BET specific surface area, pore volume, and pore size distribution. Fourier-Transform Infrared Spectroscopy (FTIR): Bruker Vector 22 spectrometer (Bruker, Karlsruhe, Germany). Samples were mixed with dry KBr at a mass ratio of 1:399 and pressed into pellets for transmission-mode measurement at ambient temperature. X-ray Photoelectron Spectroscopy (XPS): Thermo ESCALAB250 spectrometer (Thermo Fisher Scientific, Waltham, MA, USA) equipped with an Al Kα X-ray source (14 kV, 16 mA, vacuum = 4 × 10^−9^ mbar). The C 1s peak at 284.6 eV was used for charge correction; XPSPEAK software 4.1 (Hong Kong, China) was adopted for peak deconvolution and fitting. The zeta potential was measured using a ZEN3600 Malvern instrument (Malvern Panalytical, Malvern, UK) after dispersing the samples in a 0.01 mol·L^−1^ KNO_3_ solution, followed by pH adjustment with 0.01 mol·L^−1^ HNO_3_ and 0.01 mol·L^−1^ KOH. Zeta potentials at different pH values were recorded to calculate the isoelectric point (IEP) of each sample.

### 3.4. Adsorption and Cyclic Regeneration Experiments

All batch adsorption experiments were performed at ambient temperature with a constant stirring speed of 400 r·min^−1^. The initial solution pH was adjusted within the range of 3.0–11.0 using dilute HCl or NaOH. Fluoride concentrations were determined using a fluoride ion-selective electrode (F-Electrode-K2, Mettler Toledo, Urdorf, Switzerland). Prior to measurement, each sample was mixed with an equal volume of total ionic strength adjustment buffer (TISAB) to maintain constant ionic strength and pH and to eliminate interference from metal–fluoride complexation. The electrode was calibrated before each batch of measurements using a series of sodium fluoride standard solutions, and the calibration curve exhibited a correlation coefficient (R^2^) greater than 0.999.

#### 3.4.1. Adsorption Kinetic Tests

A mass of 0.1 g of each alumina adsorbent was added to 100 mL of fluoride solution with an initial concentration C_0_ = 10 mg·L^−1^. After predetermined contact durations, suspensions were filtered, and residual fluoride concentrations in the filtrates were quantified.

#### 3.4.2. Adsorption Isotherm Tests

A mass of 0.1 g of alumina was immersed in 100 mL of fluoride solution with initial concentrations of 10, 50, 100, 200, 400, and 600 mg/L, respectively. After reaching adsorption equilibrium, residual fluoride concentrations were measured to construct adsorption isotherms.

#### 3.4.3. Adsorption Mechanism Analysis

Fluoride-saturated alumina samples were collected, washed, and dried for XPS characterization. XPS spectra before and after fluoride loading were compared to reveal interfacial interactions between the alumina surface and fluoride anions.

#### 3.4.4. Cyclic Adsorption–Desorption Regeneration

A mass of 1.0 g alumina was dispersed in 1 L fluoride solution (C_0_ = 400 mg·L^−1^) and stirred for 24 h to achieve adsorption saturation. The saturated adsorbent was filtered and desorbed using a 0.1 mol·L^−1^ NaOH aqueous solution for 2 h. After desorption, the alumina was reactivated in 0.1 mol·L^−1^ HCl for 2 h, washed with deionized water until a neutral pH was reached, and subsequently dried in an oven at 140 °C for 4 h before being used in the next adsorption cycle. The above procedure was repeated for five consecutive runs.

## 4. Conclusions

Four γ-alumina adsorbents with comparable BET surface areas (180–230 m^2^·g^−1^) but distinct morphologies (nanosheet PZ-600, rhombic flake LX-600, nanofibrous XW-600, and commercial granular SB-600) were systematically evaluated for aqueous fluoride removal and cyclic reusability. The total surface hydroxyl content followed the order PZ-600 > LX-600 > XW-600, whereas the near-saturated fluoride adsorption capacity ranked PZ-600 > XW-600 > LX-600 > SB-600. These results suggest that the total surface hydroxyl content alone does not determine fluoride adsorption performance and that the accessibility and local chemical environment of surface hydroxyl species, which are influenced by alumina morphology, play an important role in fluoride adsorption. Kinetic and isotherm analyses showed that fluoride adsorption was well described by the pseudo-second-order kinetic model and the Freundlich isotherm model. XPS results were consistent with the participation of surface hydroxyl groups in fluoride adsorption through ligand exchange with fluoride ions, accompanied by electrostatic interactions, although the proposed adsorption mechanism is supported primarily by indirect evidence.

Five regeneration cycles involving NaOH desorption and HCl reactivation resulted in gradual structural evolution of the alumina adsorbents, including hydrolysis, dehydroxylation, and partial deterioration of crystallinity. Commercial SB-600 and rhombic flake LX-600 exhibited substantial structural degradation and rapid loss of adsorption capacity during cyclic regeneration. Although nanosheet PZ-600 showed the highest initial adsorption capacity, its cyclic stability was limited, which is likely associated with structural rearrangement and layer fragmentation during repeated acid–alkali treatments. In contrast, nanofibrous XW-600 exhibited superior resistance to repeated regeneration, preserving its crystal structure and fibrous morphology while maintaining the highest residual adsorption capacity among the synthesized alumina samples after five cycles. Overall, the results suggest that alumina morphology strongly influences adsorption performance and regeneration stability by affecting the structural characteristics and surface properties of the adsorbent. This work provides a facile template-free hydrothermal strategy for preparing morphology-controlled γ-alumina and offers new insights into the relationship between alumina morphology and fluoride adsorption, providing guidance for the rational design of high-performance regenerable alumina adsorbents.

## Figures and Tables

**Table 1:** BET textural parameters of as-prepared alumina samples.

Scheme	Specific Surface Area (m^2^∙g^−1^)	Pore Volume (cm^3^∙g^−1^)	Pore Size Distribution (nm)
SB-600	180.4	0.53	7.2
PZ-600	207.1	0.31	2.3
LX-600	226.5	1.18	16.2
XW-600	225.9	0.92	15.6

**Table 2:** Kinetic fitting parameters for fluoride adsorption.

Kinetic Model	Parameter	SB-600	PZ-600	LX-600	XW-600
pseudo-first-order $\frac{dq_{t}}{dt}=k_{1}\left(q_{e}-q_{t}\right)$	q_e,exp_(mg·g^−1^)	3.11	9.32	6.36	8.41
	q_e,cal_(mg·g^−1^)	3.06	9.12	5.98	6.23
	k_1_(min^−1^)	0.01971	0.00244	0.00438	0.00836
	*R* ^2^	0.96354	0.96199	0.91390	0.75668
pseudo-second-order $\frac{dq_{t}}{dt}=k_{2}{\left(q_{e}-q_{t}\right)}^{2}$	q_e,cal_(mg·g^−1^)	3.110	9.323	6.360	8.410
	k_2_(g·mg^−1^·min^−1^)	0.05377	0.00539	0.00561	0.00728
	*R* ^2^	0.99964	0.99911	0.99848	0.99840

qₜ = adsorption capacity at time t; q_e_ = equilibrium adsorption capacity; k_1_, k_2_ = rate constants; q_e,exp_, q_e,cal_ = experimental and calculated equilibrium capacity.

**Table 3:** Isotherm Fitting Parameters for Fluoride Removal.

Kinetic Model	Parameter	SB-600	PZ-600	LX-600	XW-600
Langmuir isotherm model $q_{e}=\frac{q_{m}K_{L}C_{e}}{1+K_{L}C_{e}}$	b(L∙mg^−1^)	0.0040	0.0184	0.0186	0.0161
	R^2^	0.6758	0.8356	0.9401	0.5345
Freundlich isotherm model $q_{e}=K_{f}c_{e}^{1/n}$	1/n	0.5183	0.3543	0.3416	0.2954
	K_f_ (mg∙g^−1^) (L∙mg^−1^)^1/n^	1.0815	10.8313	5.0576	5.4029
	R^2^	0.9961	0.9971	0.9982	0.9983

b = Langmuir equilibrium constant; Kf, 1/n = Freundlich characteristic constants.

**Table 4:** BET textural data of alumina after different regeneration cycles (ZS = regeneration run).

Sample	Specific Surface Area (m^2^∙g^−1^)	Pore Volume (cm^3^∙g^−1^)	Pore Size Distribution (nm)
SB-600	180.4	0.53	7.2
SB-ZS-1	170.1	0.55	7.1
SB-ZS-2	182.0	0.55	7.3
SB-ZS-3	170.0	0.54	7.1
SB-ZS-4	163.2	0.49	7.2
SB-ZS-5	143.2	0.49	7.2
PZ-600	207.1	0.31	2.3
PZ-ZS-1	276.9	0.42	2.3
PZ-ZS-2	352.2	0.56	2.4
PZ-ZS-3	406.7	0.63	3.3
PZ-ZS-4	417.4	0.73	3.8
PZ-ZS-5	410.7	0.55	2.3
LX-600	226.5	1.18	16.2
LX-ZS-1	252.5	1.23	16.3
LX-ZS-2	254.2	1.26	16.0
LX-ZS-3	258.9	1.26	16.1
LX-ZS-4	265.7	1.29	15.7
LX-ZS-5	254.8	1.13	16.0
XW-600	225.9	0.92	15.6
XW-ZS-1	231.7	1.09	16.1
XW-ZS-2	247.3	1.05	16.4
XW-ZS-3	248.1	1.05	15.8
XW-ZS-4	246.6	1.07	16.0
XW-ZS-5	244.2	1.01	15.6

**Table 5:** Comparison of representative alumina-based adsorbents for fluoride removal reported in the literature.

Adsorbent	Synthesis Method	BET Surface Area (m^2^·g^−1^)	Fluoride Adsorption Capacity (mg·g^−1^)	Regeneration Performance	Adsorption Conditions	References
SB-600	Commercial	180.4	35	0 mg·g^−1^ after 5 cycles	pH 4–8	This work
PZ-600	Template-free hydrothermal	207.1	87.2	12 mg·g^−1^ after 5 cycles	pH 4–9	This work
XW-600	Template-free hydrothermal	226.5	49	23 mg·g^−1^ after 5 cycles	pH 4–9	This work
LX-600	Template-free hydrothermal	225.9	46.1	7 mg·g^−1^ after 5 cycles	pH 4–8	This work
Nano-sized γ-alumina	surfactant-assisted solution combustion method		32	25.6 mg·g^−1^ after 5 cycles	pH 4	[44]
γ-alumina		137	16.5	5.6 mg·g^−1^ after 8 cycles	pH 4	[45]
γ-alumina	Calcination method	201.7	2.53	2.32 mg·g^−1^ after 5 cycles		[34]

## Data Availability

The original contributions presented in this study are included in this article. Further inquiries can be directed to the corresponding author.

## Supplementary Materials

The following supporting information can be downloaded at: https://www.mdpi.com/article/10.3390/molecules31152634/s1. Table S1: Summary of hydrothermal synthesis parameters for different boehmite morphologies. Figure S1: Effect of pH on adsorption of activated alumina samples with different morphologies. Figure S2: Influence of other ions on adsorption of activated alumina samples with different morphologies ((a) SB-600; (b) PZ-600; (c) XW-600; (d) LX-600).
