# Supplementary material for: Morphology-Controlled γ-Alumina Adsorbents: Fluoride Removal Performance and Cyclic Regeneration Stability in Aqueous Media
Source: Molecules. 2026 Jul 29;31(15):2634. doi: 10.3390/molecules31152634 (PMC13468667; doi:10.3390/molecules31152634)
Supplement: Supplementary file 1 [file molecules-31-02634-s001.zip › molecules-4444032-supplementary.pdf]

Supplementary Materials

Table S1. Summary of hydrothermal synthesis parameters for different boehmite morphologies.

Sample	Hydrothermal temperature (°C)	Time (h)	pH
XW	160	10	8.5-9.25
PZ	96	5	8.5
LX	160	24	9.5-10.0

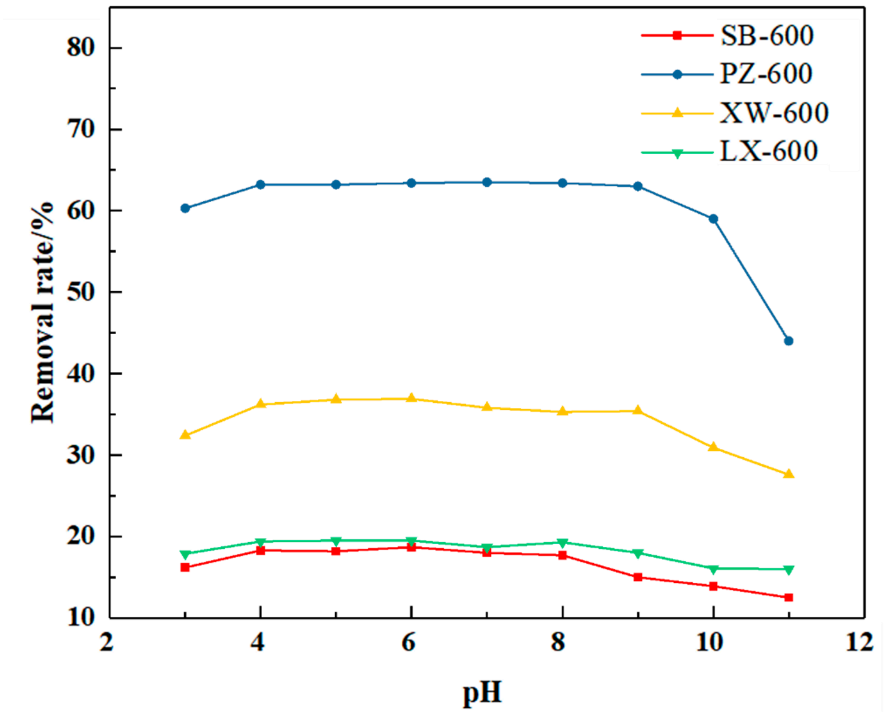

Figure S1. Effect of pH on adsorption of activated alumina samples with different morphologies

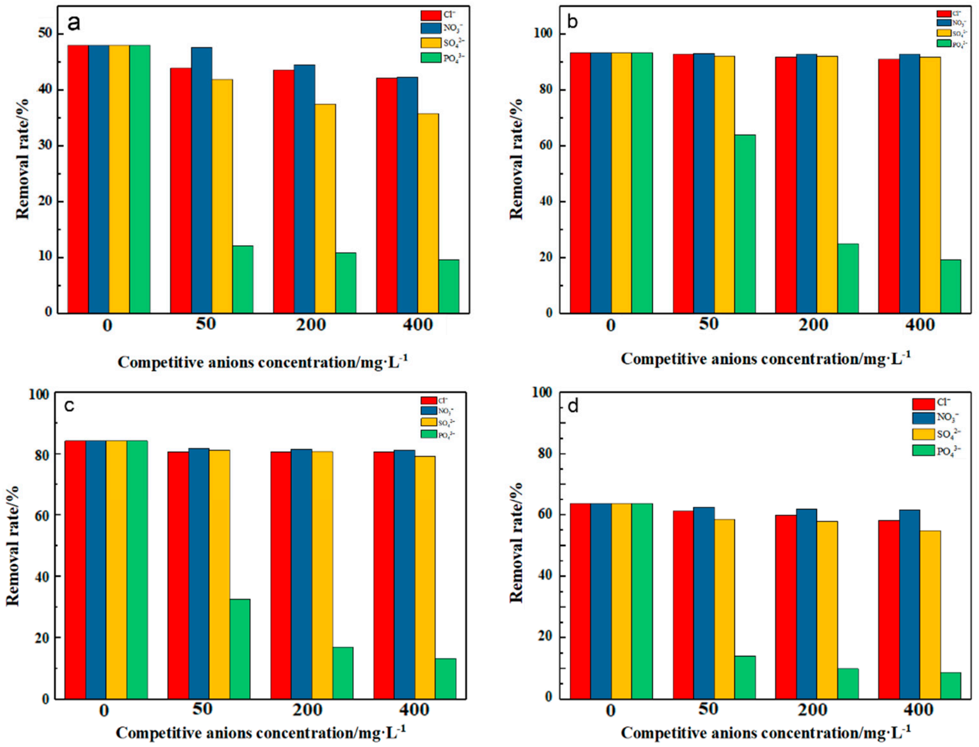

Figure S2. Influence of other ions on adsorption of activated alumina samples with different morphologies ((a) SB-600; (b) PZ-600; (c) XW-600; (d) LX-600)
